# Paraneoplastic Necrotizing Myopathy Associated With Metastatic Colon Cancer: A Case Report

**DOI:** 10.7759/cureus.36538

**Published:** 2023-03-22

**Authors:** Sethi Ashish, Moses Raj, Mohammad Islam, Eric Zhuang

**Affiliations:** 1 Medical Oncology, University of Missouri Kansas City School of Medicine, Kansas City, USA; 2 Hematology and Medical Oncology, Allegheny Health Network, Pittsburgh, USA

**Keywords:** cancer-associated myositis, wild-type kras, metastatic colon cancer, dermatomyositis, polymyositis, folfox, cetuximab, necrotizing myopathy, paraneoplastic syndromes

## Abstract

Necrotizing myopathy (NM) as a paraneoplastic process in malignancies is a rare phenomenon. An association of inflammatory myositis with malignancy and chemotherapies has been reported in several case reports. Here, we present an unusual case of paraneoplastic NM associated with metastatic colon cancer.

## Introduction

Some patients develop a parallel relation between cancer and myositis [1]. Dermatomyositis (DM) and seronegative immune-mediated necrotizing myopathy (IMNM) are the two most common subtypes of inflammatory myositis seen in malignancies [2]. Myositis due to chemotherapies or as immune-mediated adverse events have been reported in the medical literature. Here, we present an unusual case of paraneoplastic necrotizing myopathy (NM) associated with metastatic colon cancer.

## Case presentation

**Figure 1:**
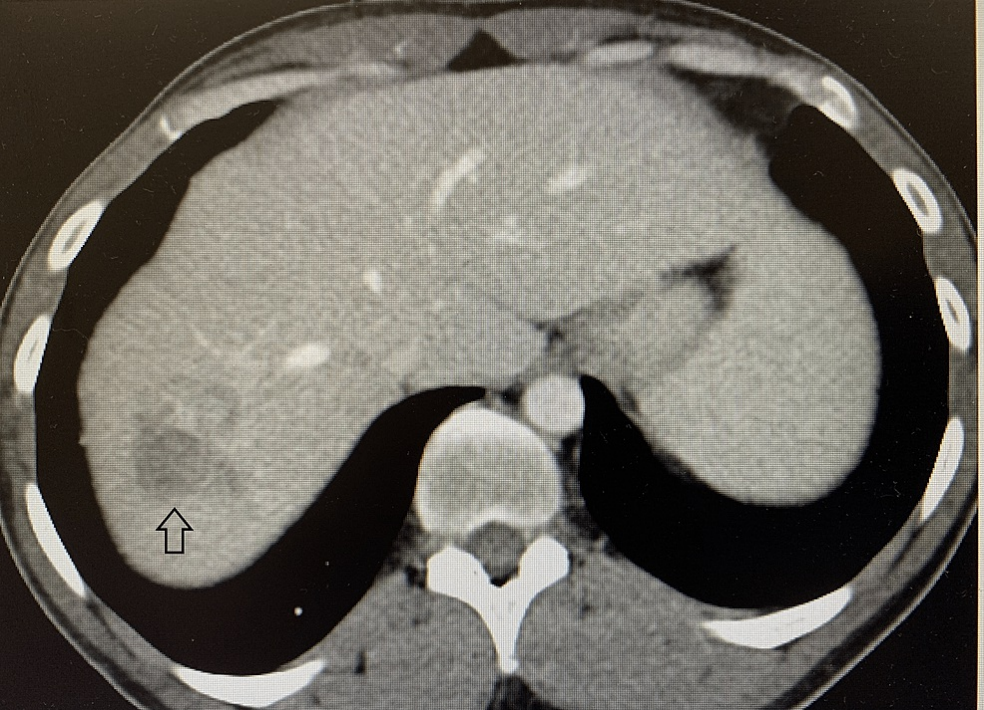
Computed tomography scan illustrating liver mass (black arrow)

**Figure 2:**
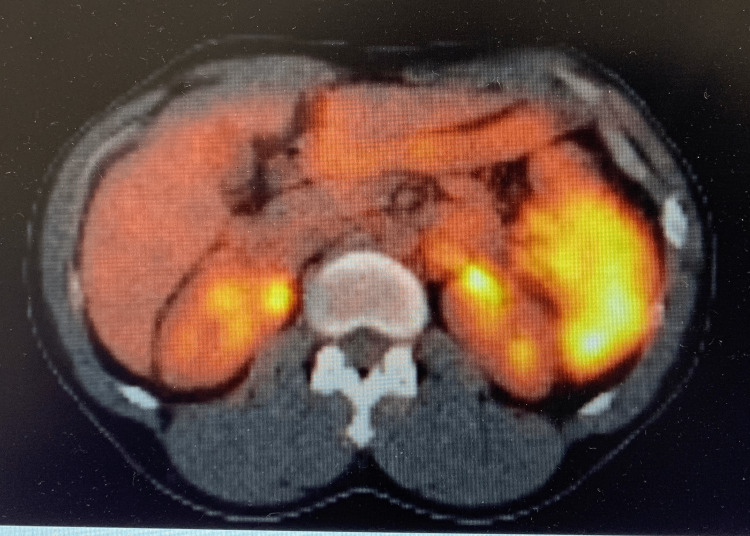
PET scan illustrating hypermetabolic left splenic flexure mass PET - positron emission tomography

**Figure 3:**
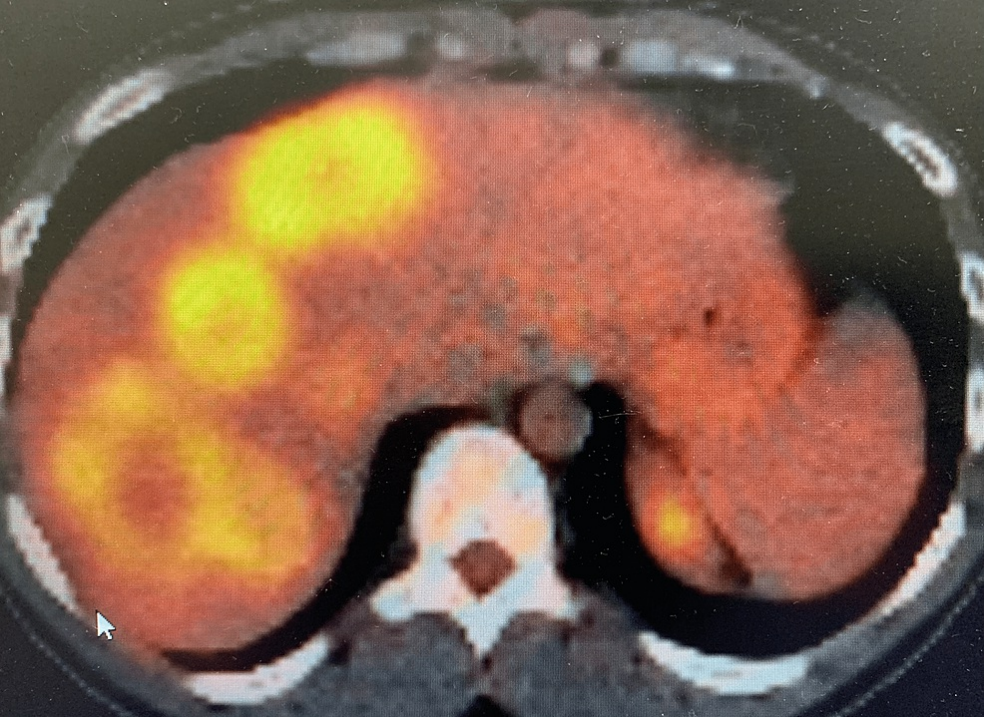
PET scan illustrating multiple hypermetabolic metastases in the liver PET - positron emission tomography

A 35-year-old healthy male complained of acute abdominal pain, nausea, and vomiting. Computed tomography (CT) of the abdomen and pelvis revealed a large obstructing splenic flexure mass with resultant upstream large bowel obstruction of transverse and right hemicolon. The left upper quadrant with surrounding pericolonic and left retroperitoneal lymphadenopathy revealed an extraluminal extension of the mass with subadjacent soft tissue masses. Further, numerous large hepatic masses were evident on the CT scan illustrating metastases (Figure 1). The patient underwent-diverting loop transverse colostomy and liver biopsy, which confirmed stage IV metastatic colorectal adenocarcinoma. Positron emission tomography (PET) scan also showed a hypermetabolic left splenic flexure mass with multiple adjacent left upper quadrant soft tissue masses and mesenteric lymph nodes, left para-aortic retroperitoneal node lymphadenopathy (Figures 2, 3) with multiple hypermetabolic metastases in the liver. The patient was initially treated with folinic acid, fluorouracil, and irinotecan (FOLFIRI), and later, oxaliplatin was added to the chemotherapy treatment. Molecular testing or next-generation sequencing revealed Kirsten rat sarcoma viral oncogene homolog (KRAS) wildtype; thus, cetuximab was added with cycle three of modified folinic acid, fluorouracil, irinotecan, oxaliplatin (FOLFIRINOX). The patient's treatment with an anti-epidermal growth factor receptor (EGFR) inhibitor, cetuximab was continued, but other chemotherapies were held for the plan of hepatic artery infusion (HAI) pump.

**Figure 4:**
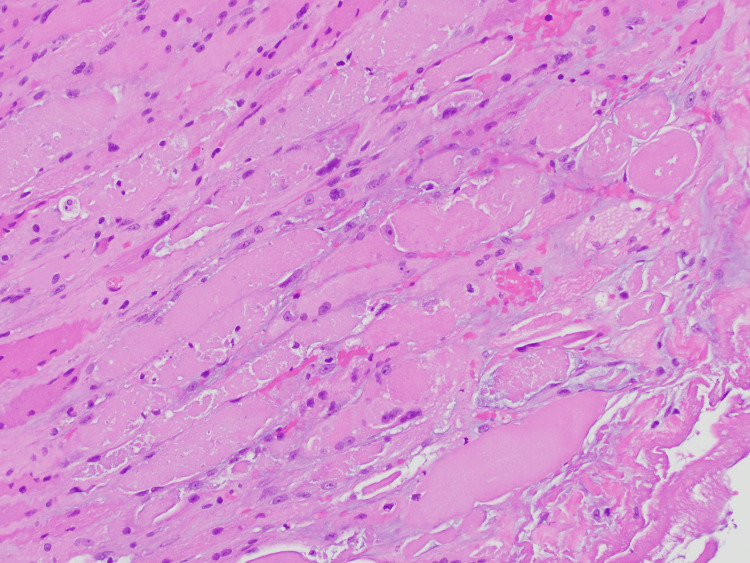
Hematoxylin and Eosin stain: necrotizing myopathy Stained frozen sections show moderate variation in fiber diameter, with prominent necrotic fibers associated with a neutrophilic infiltrate. There is a mild increase in internal nuclei and rare ring fibers are seen. Scattered nuclear bags are also present. A prominent chronic inflammatory infiltrate is not seen and there is no evidence of vasculitis. There is no fibrosis; however, edema is seen. Rare rimmed vacuoles are seen.

**Figure 5:**
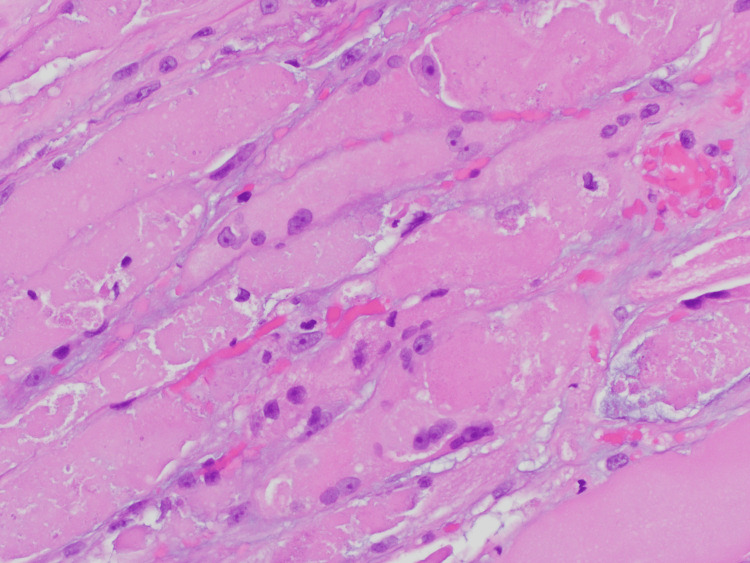
Hematoxylin and Eosin stain: necrotizing myopathy high power (40x)

Meanwhile, the patient was admitted for rhabdomyolysis/myositis with creatine kinase levels rising up to 12,751 units per liter (U/L). C-reactive protein (CRP) was up to 19.2 mg/dL, and the erythrocyte sedimentation rate (ESR) was up to 128 mm/hr. He was promptly started on intravenous (IV) fluid hydration. A comprehensive serologic work-up was performed, which revealed negative antibodies to amphiphysin, anti-glial nuclear type 1, anti-neuronal nuclear type 1, anti-neuronal nuclear type 2, anti-neuronal nuclear type 3, crmp-5 IgG, neuronal K+ channel, calcium channel P/Q, Purkinje cell cytoplasm type 1, Purkinje cell cytoplasm type 2, Jo-1, PL7, PL12, EJ, OJ, SRP, MI2, MDA-5, NXP-2, SAE-1, PM-SCL, SSA, SSB, U1 -RNP, U2-RNP, U3-RNP, and histone. He was also negative for rheumatoid factor and antinuclear antibody (ANA). He was positive for anti-TIF-1 gamma with a titer of 120 units, with values >80 considered strong positive. His case was discussed in a multi-disciplinary conference, including medical oncology, surgical oncology, radiology, and rheumatology. The differential diagnosis included paraneoplastic myopathy, polymyositis, and chemotherapy-associated myopathy. The consensus was to initiate empiric corticosteroids, hold cetuximab, and re-rechallenge systemic chemotherapy, starting with one drug at a time with the goal of reaching triple agent therapy with modified FOLFIRINOX. A couple of weeks later, he resumed chemotherapy, starting with 5-FU after allowing for the patient's performance status to improve. Given the suspicions of an underlying paraneoplastic process driving the myositis, the idea was that systemic therapy would improve his overall disease burden and induce a remission of the myositis. The patient tolerated single chemotherapy agent additions well and eventually reached triplet therapy with modified FOLFIRINOX, with no exacerbation of myositis. His symptoms of myositis completely resolved, and his oral steroids were tapered over the course of two to three weeks. However, after completion of the steroid taper, the patient developed a relapse of myositis and was admitted again. The clinical timeline favored an alternative diagnosis to cetuximab-associated myositis as he had been off of the drug for over a month. Additionally, because he had tolerated single agent additions of chemotherapy so well and that his symptoms reappeared rapidly (within a week), this raised the suspicion for a paraneoplastic process as the underlying etiology. It was also felt that his steroid regimen was tapered too quickly. In order to further evaluate his myositis, he underwent a random muscle biopsy of the right vastus lateralis, which demonstrated necrotizing myopathy (Figures 4, 5). The patient was then restarted on corticosteroids, leading to rapid improvement in his all four-extremity muscle weakness. He was initially started on 1 mg/kg of glucocorticoids with 80 mg prednisone which was subsequently slowly tapered every week over the course of eight weeks until the completion of all doses. After the acute episode of myositis improved, chemotherapy was resumed a couple weeks later and he had no further episodes of myositis on last follow-up nine months later. 

## Discussion

Paraneoplastic necrotizing myopathy has been described as a rapidly progressive variant of inflammatory myopathies associated with a malignancy affecting adults more than 40 years of age [3]. Chemotherapy-associated myopathy has been described in the literature and must also be on the differential in a patient receiving treatment who presents with myopathy. Docetaxel, a commonly used cytotoxic chemotherapy agent used in various cancer subtypes, has been associated with myalgia-arthralgia syndrome, characterized by severe generalized musculoskeletal pain [4]. Similarly, Florczynski et al. reported a case of severe myopathy two months after the completion of neoadjuvant treatment with FOLFOX for rectal adenocarcinoma [5]. In contrast to necrotizing myopathy, autoimmune necrotizing myopathy is commonly seen in autoimmune disorders such as scleroderma and mixed connective tissue disorder and can also be triggered by statin therapy. Statin-induced myopathy evolves into an autoimmune myopathy that progresses beyond three months after statin discontinuation in predisposed individuals. The onset of autoimmune necrotizing myopathy may be seen as late as 10 years following the start of statin therapy [6]. Like in autoimmune necrotizing myopathy, high-dose corticosteroids are considered as first-line treatment for cancer-associated myositis or paraneoplastic myopathy. Most cases with paraneoplastic myopathy are also resistant to corticosteroids. Immunosuppressive therapy with methotrexate and azathioprine can be used for the treatment of paraneoplastic myopathy. Additionally, intravenous immunoglobulin or plasmapheresis can also cause favorable outcomes [7].

Paraneoplastic syndromes like Eaton-Lambert syndrome, limbic encephalitis, and cerebellar ataxia are mediated by cancer-associated antibodies and are usually linked with particular cancer types [8]. Identification of an association with specific cancer types would both enhance our understanding of the cause of myositis and help to identify focused diagnostic workup for cancer screening in different patients [9]. Alternatively, immunotherapy-induced myositis patients have characteristic clinicopathological phenotypes combining limb-girdle, axial, and oculomotor weakness with a unique pattern of pseudo-granulomatous necrotic infiltrates of macrophages and T cells. Patients with immunotherapy-induced myositis may also develop myocarditis [10, 11]. Differentiating IMNM of paraneoplastic origin from IMNM induced by immunotherapy is challenging but important, as the management of the latter would require dose modification or even discontinuation of the offending agent [1].

Dropped head syndrome is caused by weakness of the neck extensors, while most myopathies involve the proximal muscle of the extremity with elevated creatine kinase. The syndrome has been observed after exposure to selumetinib, which is used to treat melanoma and other solid tumors. It causes a focal non-inflammatory myopathy characterized by neck pain and weakness of neck extensors [12]. Steroids are not useful in managing this syndrome and can increase myopathic symptoms because this is a non-inflammatory process. Drug discontinuation is the best way to resolve this form of myopathy [12]. In our case, we decided to rechallenge the patient with chemotherapy, adding back one agent at a time, in order to help reduce the disease burden and consequentially induce myositis remission, given the suspicions of an underlying paraneoplastic process. 

## Conclusions

Necrotizing myopathy has a poor prognosis if the underlying etiology is not promptly diagnosed and appropriately managed. Paraneoplastic necrotizing myopathy is a rare phenomenon; however, it has been described to be associated with colon cancer and anti-TIF1 gamma antibodies. The differential diagnoses for proximal muscle weakness due to myopathy are broad and include polymyositis, dermatomyositis, inclusion body myositis, and drug-associated myopathy. High-dose corticosteroids tapered over a long course, in addition to management of the underlying malignancy, are an effective treatment for malignancy-associated necrotizing myopathy. Further studies are necessary to elucidate the underlying associations between different malignancies and paraneoplastic myopathy, along with optimal management. 
